# A novel missense mutation of the *STK11* gene in a Chinese family with Peutz-Jeghers syndrome

**DOI:** 10.1186/s12876-022-02617-y

**Published:** 2022-12-22

**Authors:** Zhen Yu, Lin Liu, Fang Jiang, Yimin Ji, Xiao Wang, Lili Liu

**Affiliations:** 1 Department of Pediatrics, Qilu Hospital, Shandong University, 107 Wen Hua Xi Road, Jinan, 250012 Shandong People’s Republic of China; 2 Shandong Provincial Maternal and Child Health Care Hospital, Shandong University, 238 Jing Shi Dong Road, Jinan, 250012 Shandong People’s Republic of China

**Keywords:** Mutation, Peutz-Jeghers syndrome, *STK11*, Gastrointestinal polyps

## Abstract

**Background:**

Peutz-Jeghers syndrome (PJS) is a rare autosomal dominant inherited disease caused by mutations in the Serine-Threonine Kinase 11 (*STK11*) gene. This study aimed to diagnose a Chinese pedigree with PJS and to expand the spectrum of *STK11* variants.

**Methods:**

We performed an inductive analysis of clinical features, gastrointestinal endoscopy, radiologic imaging, and pathological findings in a Chinese family with PJS. Whole-exome sequencing (WES), Sanger sequencing, and *STK11* protein 3D structure prediction were performed for establishing a molecular diagnosis.

**Results:**

The proband, her mother, and grandfather presented with pigmentation spots on lips, oral mucosa, and fingers. Her mother and grandfather also had pigmentation spots on face and feet, while her brother had pigmentation spots only on the lower lip. On endoscopy, polyps were discovered in the proband, her mother, and grandfather. A novel heterozygous mutation (c.521A > C) in exon 4 of *STK11* was identified in all four patients, leading to a change from histidine to proline in amino acid 174. The variable site p.H174 was highly conserved in different species on multiple sequence alignment analysis.

**Conclusions:**

We diagnosed a Chinese pedigree with PJS based on clinical features, gastrointestinal endoscopy, and genetic testing results. Our results expanded the spectrum of *STK11* variants, which will be helpful for genetic counseling.

## Background

Peutz-Jeghers syndrome (PJS) is a rare autosomal dominant inherited disease caused by mutations in the Serine-Threonine Kinase 11 (*STK11*) gene. The diagnosis of PJS is based on the clinical manifestations and the World Health Organization (WHO) criteria for the syndrome [1]. The clinical features of PJS include mucocutaneous pigmentation, gastrointestinal hamartomatous polyps, and an increased risk of predisposition toward developing malignancy [2, 3]. The disorder is discovered in various ethnic groups; it shows no differences between genders, and it has an unknown incidence estimated to range from 1:50,000 to 1:200,000 [4, 5]. Unfortunately, large-scale epidemiological studies on PJS have not been performed in Chinese. Li Meng et al. collected peripheral blood of 64 PJS patients from January 2016 to August 2018 for an STK11 gene mutation test, revealing 39 types of mutations consisting of missense, nonsense, insertional, deletional, and splice mutations [6]. Frameshift, synonymous, and intronic mutations were also identified in Chinese [7]. The polyps can lead to complications, including abdominal pain, intussusception, intestinal obstruction, prolapse, and gastrointestinal bleeding [3].

Germline mutations in the *STK11* gene at chromosome 19p13.3 have been identified as a major cause of PJS [8, 9]. *STK11* was previously known as Liver Kinase B1 (*LKB1*) [8]. It consists of nine coding exons and one non-coding exon, encoding a 433 amino acid serine-threonine protein kinase [8, 9]. *STK11* is a tumor suppressor that acts as an early gatekeeper [10]. *STK11* forms a heterotrimeric complex with STRAD and MO25 in the cytoplasm and undergoes conformational changes to result in autophosphorylation [11]. It regulates cell polarity [12], and it controls cell cycle arrest, cell proliferation, and apoptosis [13].

## Methods

### Ethical approval

The study was approved by the Ethics Committee of Qilu Hospital of Shandong University. The methods were carried out in accordance with the approved guidelines.

### Patients and sample collection

Kindred subjects were diagnosed with PJS based on the WHO criteria, and they received polyp surgery in Qilu Hospital of Shandong University. The subject family with PJS participated in this study after providing their informed consent in October 2021. Peripheral blood samples were obtained from all individuals, and comprehensive clinical data, such as medical history, pedigree, physical examination, and endoscopic and pathological findings, were collected. Intestinal polyp specimens were obtained from the proband, her mother, and her grandfather.

### Exome sequencing and variant detection

Genomic DNA was isolated from peripheral blood of the proband, her brother, and her parents. WES was performed by MyGenostics (Beijing, China) using the Illumina HiSeq X Ten system. The FASTQ files were mapped to the reference human genome (hg19) and then assessed for variant calling using the HaplotypeCaller tool of GATK software.

### Bioinformatics analysis

The potential deleterious effects of the missense mutation in *STK11* were predicted using in silico algorithms provided by the online software PolyPhen-2 score (http://genetics.bwh.harvard.edu/cgi-bin/pph2), SIFT (http://sift.bii.a-star.edu.sg/sift-bin/), MutationTaster (http://www.mutationtaster.org/cgi-bin/), and GERP++ (http://mendel.stanford.edu/SidowLab/downloads/gerp/index.html). Diagnostic variants were defined as pathogenic or likely pathogenic according to the guidelines of the American College of Medical Genetics and Genomics (ACMG) [14]. Evidence for disease causality was assessed using ClinVar (https://www.ncbi.nlm.nih.gov/clinvar/). To confirm the conservation of amino acid substitutions in the process of species evolution, the typical protein sequences of multiple different species were aligned using UGENE software to compare mutated positions with conserved domains. Structure prediction was performed by the Swiss-Model online software (https://swissmodel.expasy.org/). Identified variants were confirmed by Sanger sequencing. The primers used to target human *STK11* included forward: 5′-TGCCTGGACTTCTGTGACTTC-3′ and reverse: 5′-CCAGATGTCCACCTTGAAGC-3′.

## Results

### Clinical manifestations

**Fig. 1:**
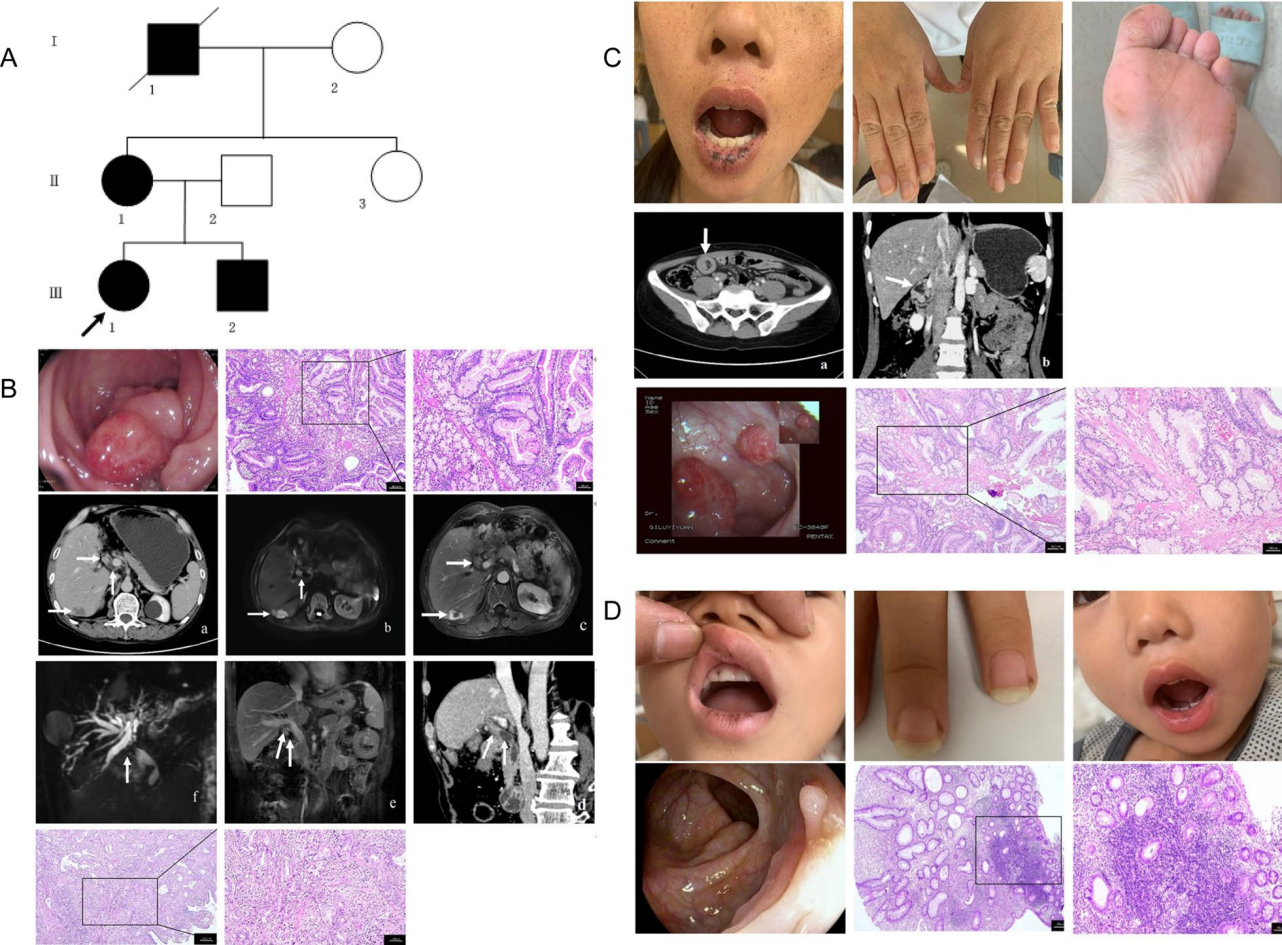
A genogram and clinicopathological features of the subject family with PJS.** A** Pedigree of the family with PJS. Roman numerals indicate generations and Arabic numbers indicate individuals. Squares = males, and circles = females. Filled and unfilled symbols denote affected and unaffected individuals, respectively. Slash indicates decedent. The proband is indicated by an arrow. **B** Endoscopic, histopathological, and radiologic imaging signs exhibited by the proband’s grandfather. Upleft, endoscopy showed polyps in the sigmoid colon; upper right, representative hematoxylin–eosin-stained tissue slices of the polyp specimens confirm hamartomatous polyps (left, × 40 magnification; right, × 100 magnification); middle, contrast enhanced CT and MRI, MRCP of lesions in the liver; bottom, well-differentiated adenocarcinoma in the hilar bile duct, nerve invasion, and focal invasion into the liver tissue were consistent with the pathology of cholangiocarcinoma (left, × 40 magnification; right, × 100 magnification). **C** PJS signs exhibited by the proband’s mother. Up, pigmentation on the lips, face, fingers, and feet; middle, contrast enhanced CT showed that the small intestine and mesentery gathered in a round shape, presenting a ‘target sign’; MPR showed that two moderately uneven and round enhanced masses were seen in the duodenal cavity; bottom, gastrointestinal tract polyps of the sigmoid colon and histopathology (left, × 40 magnification; right, × 100 magnification). **D** Signs exhibited by the proband and her brother. Upleft, pigmentation on the proband’s lips, oral mucosa, and fingers; upper right, pigmentation on the lower lip of the proband’s brother; bottom left, gastrointestinal endoscopy images showed polyps in the ascending colon; bottom right, histology of the resected polyps in the proband, with features of a juvenile polyp (left, × 40 magnification; right, × 100 magnification)

Four patients distributed across three generations exhibited an autosomal dominant mode of inheritance (Fig. 1A). The age of onset of mucocutaneous pigmentation was as early as one year in II1, and two years in III1 and III2 (Table 1; Fig. 1B, C and D).

**Table 1:** Clinical characteristics of the affected family members

ID	II1	III1	III2	I1
Gender	Female	Female	Male	Male
Age (years)	31	6	3	57
Age at onset of mucocutaneous pigmentation (years)	Earlier than 1 year	Earlier than 2 years	Earlier than 2 years	Unknown
Order of onset of mucocutaneous pigmentation	Lip, followed by fingers, feet, oral mucosa, and face	Lip, followed by fingers and oral mucosa	Lower lip	Unknown
Age at the first occurrence of intestinal intussusception/obstruction	21	No	No	15
Age at the first diagnosis and resection of polyps	21	6	No	15
Location of polyps	Duodenum, ileocecal region, ascending colon, transverse colon, descending colon, sigmoid colon, and rectum	Ascending colon and rectum	No	Stomach, duodenum, ileocecal region, ascending colon, transverse colon, descending colon, sigmoid colon, and rectum

I1 had mucocutaneous pigmentation on the lips, face, fingers, toes, and oral mucosa since early childhood. At the age of 15, the patient suffered from intestinal polyps and obstruction and received intestinal surgery at a local hospital. At the age of 50, he presented with a 6-year-history of hematochezia and underwent endoscopy, during which multiple polyps of the colon and rectum were discovered (Fig. 1B). The patient was diagnosed with PJS after pathological examination of the polyps (Fig. 1B) and received surgery for nasopharyngeal cancer in the same year. All resected polyps showed hamartomatous histopathology with peculiar proliferation of smooth muscle cells, growing in a branching-tree pattern and extending into the lamina propria with displacement of the surface epithelium into the submucosa and muscularis propria (Fig. 1B). At the age of 55, he underwent ileocecal resection and ileostomy because of intussusception from polyps. At the age of 57, 19 polyps were resected from the stomach and duodenum. An enhanced computed tomography (CT) examination was performed before surgery, which demonstrated an irregular enhanced mass in the common bile duct with dilation of the bile ducts. A diagnosis of biliary tumors was considered. Magnetic resonance imaging (MRI), magnetic resonance cholangiopancreatography (MRCP), and multiplanar reconstruction (MPR) performed after surgery revealed multiple lamellar long T1 and slightly longer T2 signals in the liver, and a low signal filling defect area in the common bile duct of the liver hilus with a dilated intrahepatic bile duct. A peripheral rim of nodular enhancement was observed in the arterial phase, followed by progressive filling enhancement during the portal venous phase and delayed phase. The upper segment of the common bile duct was thick. The bile ducts were obviously dilated. The walls of the bile duct in the hilar region were thickened, with high signal intensity on diffusion-weighted imaging, and obvious enhancement and delayed enhancement on an enhanced scan (Fig. 1B). He underwent an operation for advanced cholangiocarcinoma (Fig. 1B) later, and died 5 months after surgery.

II1 was a 31-year-old female patient who had been known to have PJS since 2010. The diagnosis was based on familial history of PJS, the presence of characteristic mucocutaneous pigmentation (Table 1), and PJ polyps. She suffered from diffuse abdominal pain and was diagnosed with intussusception by abdominal ultrasonography and CT in 2010. Emergency resection with anastomosis was performed. After that, she underwent endoscopic polypectomy four times from 2012 to 2019, and during this period, she experienced a recurrence of intussusception in December 2019 (Fig. 1C). Resected pedunculated polyps, ranging from 0.3 to 3 cm in diameter, were found in the duodenum, ileocecal region, colon, and rectum (Fig. 1C; Table 1). Postoperative pathology showed characteristics of typical PJ polyps, which was the same as that in her father (Fig. 1C).

III1 was diagnosed with PJS because of familial history of PJS and mucocutaneous pigmentations on the lips, fingers, and oral mucosa, but not on the toes (Table 1). Two polyps were resected from the cavity of the ascending colon and rectum during endoscopic examination. The smaller polyp measured 0.3 × 0.4 cm, and it was covered by red smooth mucosa on the surface; the larger polyp measured 0.5 × 0.6 cm, and it appeared as polypoid hyperplasia with a sessile, rough, and loculated surface. Pathology showed a hamartomatous polyp with normal epithelium and an inflammatory infiltrate with dilated, mucus-filled cystic glands in the lamina propria, consistent with a juvenile polyp (Fig. 1D).

III2 had mucocutaneous pigmentation on the lower lip, but not on the fingers or toes; meanwhile, polyps were not detected in his gastrointestinal tract (Fig. 1D; Table 1).

The proband’s grandmother and aunt were healthy, and they did not have mucocutaneous pigmentation.

### Mutation detection

**Fig. 2:**
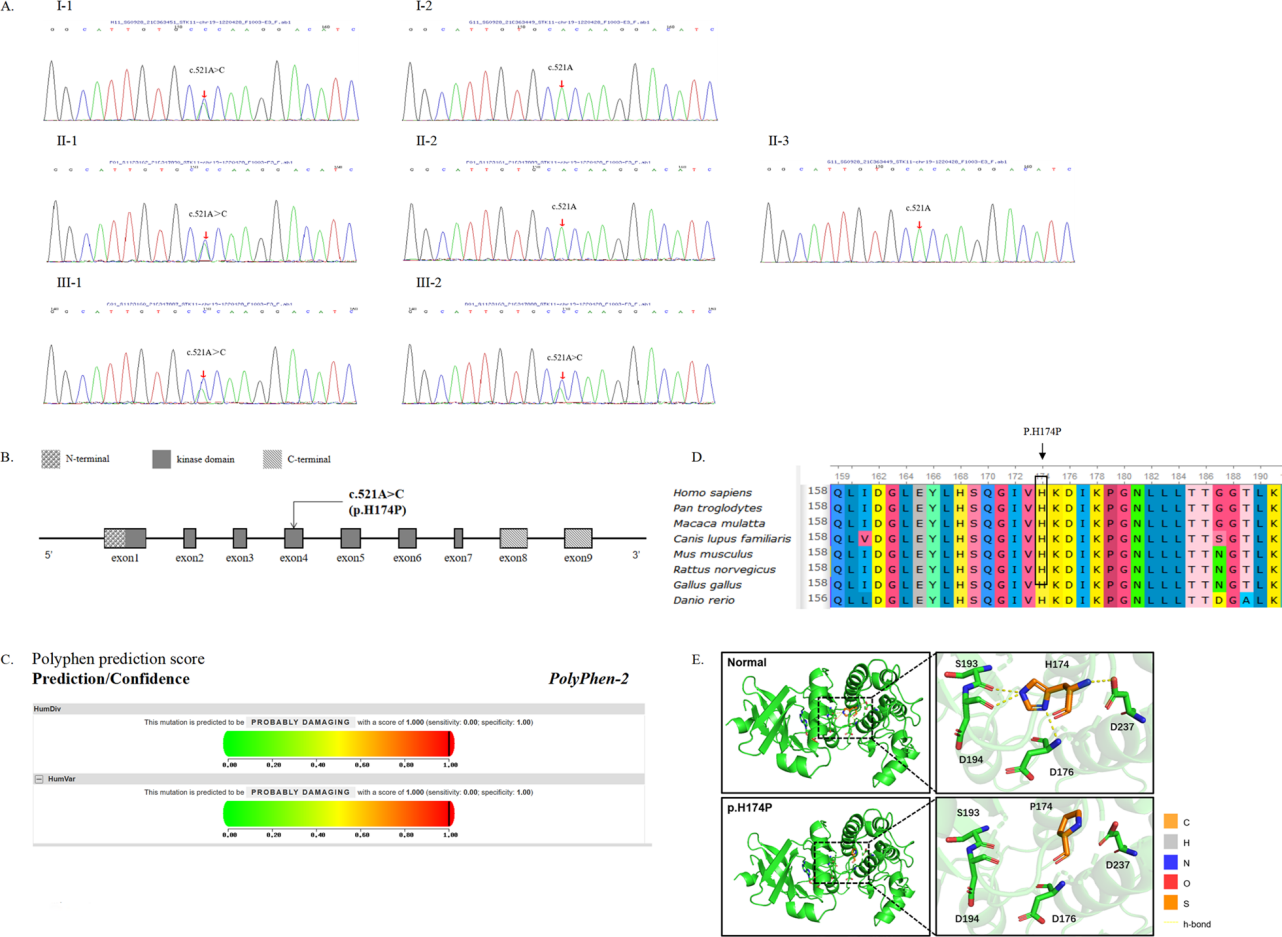
Sequencing results and bioinformatic analysis of the gene mutation. **A** Sanger sequencing results of the variants. A heterozygous mutation c.521 A > C transition, causing the substitution of histidine by proline at codon 174 (NM_000455). **B** The gene structure of *STK11*. c. 521 A > C (p.H174P) is located in exon 4 within the kinase domain. **C** Score of the novel damaging mutation c.521 A > C (p.H174P) in PolyPhen-2. **D** Evolutionary conservation showed that the variable site p.H174P was highly conserved across different species. **E** Protein structure prediction of wild-type and mutant *STK11* is displayed

To further confirm the diagnosis, we subsequently applied WES in the proband and her family members. A heterozygous missense variant c.521A > C of *STK11* was identified in patients III1, III2, II1, and I1 (Fig. 2A). The variant led to the replacement of histidine to proline in codon 174 (p.H174P, Fig. 2B).

Bioinformatic prediction was performed for the mutation, and it yielded a PolyPhen-2 (http://genetics.bwh.harvard.edu/pph2/) score of 1.000, indicating that it was possibly damaging (Fig. 2C), and it also yielded a SIFT (http://sift-dna.org/sift4g) score of 0, indicating that it affected protein function. The amino acid in this residue was evolutionarily conserved (Fig. 2D), which indicated an important functional role. The 3D structures of the mutated protein constructed using the Swiss-model (http://swissmodel.expasy.org) showed loss of the hydrogen bond in the local structure (Fig. 2E) [15]. In addition, we confirmed that this novel mutation was likely pathogenic in these patients with PJS according to the guidelines of the American College of Medical Genetics and Genomics (ACMG) [14], and it broadened the spectrum of *STK11* variants associated with PJS. This mutation has not been reported in literatures or recorded in the Human Genome Mutation Database (HGMD, http://www.hgmd.cf.ac.uk/), suggesting that it was a novel variation. The variable site was confirmed and was also identified in her brother, her mother, and her grandfather by Sanger sequencing (Fig. 2A).

## Discussion

Peutz-Jeghers Syndrome, an autosomal dominant hereditary polyposis syndrome, has various manifestations associated with gastrointestinal polyps, such as abdominal pain, hematochezia, chronic anemia, prolapsed rectal polyp, intussusception, and bowel obstruction [16–18]. Patients with PJS have an inherited predisposition to gastrointestinal malignancies [19] and other cancers, such as gynecological, testicular, breast, pancreatic, lung, and thyroid papillary cancers [20, 21]. In our study, older patients were often prone to develop serious outcomes, leading to inevitable open surgery or even cancer. Patient I1 presented with the classic PJS-phenotype with pigmentation and intestinal obstruction. However, only at the age of 50, he was diagnosed with PJS based on mucocutaneous pigmentation and findings of endoscopy and histopathology. Then he underwent ileocecal resection and ileostomy because of intussusception from polyps and he underwent an operation for nasopharyngeal cancer and cholangiocarcinoma. At the age of 57, he died due to advanced cholangiocarcinoma, 5 months after surgery. Patient II1 also had typical manifestations of PJS and received emergency resection and anastomosis because of intussusception in 2010. After that, she underwent endoscopic polypectomy four times. Fortunately, the tragedy increased the awareness about the disease condition and allowed timely diagnosis and treatment of the children (III1 and III2). We identified a likely pathogenic mutation in *STK11*, providing molecular diagnostic evidence for PJS.

As a tumor suppressor gene, *STK11* located on chromosome 19p 13.3, and variants of *STK11* were confirmed as the key determinants of PJS [22]. Hundreds of mutations in the *STK11* gene have been reported to be related to PJS [23]. Resta et al. reported that truncating variants trended towards early-onset cancer than missense variants in *STK11* [24]. However, other researchers reported that there was no statistically significant difference in the phenotype between patients who had PJS with truncating and missense variant types [25–28]. *STK11* variant locations were found to show a non-random distribution and missense variants were overexpressed in exons 4 and 7 [29]. In this study, we employed WES to detect mutations in a Chinese family with PJS. A novel missense mutation (c.521A > C, p.H174) in exon 4 of *STK11* was identified in patients I1, II2, III1, and III2, which was predicted to cause damaging consequences, analyzed by SIFT, PolyPhen2, and Mutation Taster. The variation was verified by Sanger sequencing. The mutation was not present in healthy family members. Co-segregation of the mutation and disease indicated that this was a pathogenic mutation. Analysis with UGENE software revealed that histidine at position 174 was highly conserved in different species, suggesting that it is structurally and functionally important. To study the functional affection of the mutation, we investigated the three-dimensional structure of STK11 c.521A > C (p.H174P) by the Swiss-Model online software (https://swissmodel.expasy.org/), and it showed that the hydrogen bonds formed by amino acid 174 and amino acid 193, 194, 176, and 237 had disappeared and the strength was lower.

In general, mucocutaneous pigmentation is usually the earliest clinical manifestation in patients with PJS. However, it is easily overlooked or misdiagnosed as nevi, and it tends to fade after puberty or at an older age, which should be taken into consideration when registering a family history [30]. Sometimes, PJS is not diagnosed until severe symptoms develop and emergency surgery is required. Genetic testing is an effective way for early diagnosis and genetic counseling of individuals whose family has a positive history. If they conceive, prenatal genetic testing could help them prevent the birth of an affected baby based on the identified pathogenic mutation. In fact, prenatal diagnosis of PJS by genetic testing of *STK11* has been successfully performed in India [31] and China [32].

According to the current recommendations, patients with PJS should receive endoscopy every 3 years until the age of 50 [3]. Well-timed polypectomy is necessary to avoid possible gastrointestinal complications and high cancer risk throughout the patient’s lifetime.

## Conclusions

In this study, we diagnosed a Chinese pedigree with PJS based on the clinical data, radiographic features, and genetic testing results. We provided the information necessary to improve the early diagnosis of PJS and further broaden the genetic mutation spectrum of *STK11*.

## Data Availability

The datasets generated and/or analysed during the current study are not publicly available due the Chinese policy of “National regulation on the management of human genetic resources” released by State Council (Index No. 000014349/2019-00063; Serial No. 171)] but are available from the corresponding author on reasonable request.

## Abbreviations

*STK11*: Serine-Threonine Kinase 11
PJS: Peutz-Jeghers syndrome
WES: Whole-exome sequencing
WHO: The World Health Organization
*LKB1*: Liver Kinase B1
hg19: Human genome
ACMG: The American College of Medical Genetics and Genomics
CT: Computed tomography
MRI: Magnetic resonance imaging
MRCP: Magnetic resonance cholangiopancreatography
MPR: Multiplanar reconstruction
DWI: Diffusion-weighted imaging
HGMD: The Human Gene Mutation Database
